# Daclatasvir/asunaprevir based direct-acting antiviral therapy ameliorate hepatitis C virus-associated cryoglobulinemic membranoproliferative glomerulonephritis: a case report

**DOI:** 10.1186/s12882-017-0534-5

**Published:** 2017-03-29

**Authors:** Michiko Shimada, Norio Nakamura, Tetsu Endo, Hideaki Yamabe, Masayuki Nakamura, Reiichi Murakami, Ikuyo Narita, Hirofumi Tomita

**Affiliations:** 1 Department of Cardiology and Nephrology, Hirosaki University Graduate School of Medicine, 5 Zaifu-cho, Hirosaki, Japan 036-8562; 2 Community Medicine, Hirosaki University Graduate School of Medicine, Hirosaki, Japan; 3 Department of Gastroenterology and Hematology, Hirosaki University Graduate School of Medicine, Hirosaki, Japan

**Keywords:** Membranoproliferative glomerulonephritis, Cryoglobulinemia, HCV-associated glomerulonephritis, Direct-acting antivirals

## Abstract

**Background:**

Direct-acting antivirals (DAAs) dramatically improve the treatment of hepatitis C virus (HCV) infections. However, the effects of DAAs on extra-hepatic manifestations such as HCV-associated glomerulonephritis, especially in cases with renal dysfunction, are not well elucidated.

**Case presentation:**

A 69-year-old Japanese woman was diagnosed as having chronic hepatitis C, genotype 1b at the age of 55. She presented with hypertension, microscopic hematuria, proteinuria, renal dysfunction, purpura, and arthralgia at the age of 61. She also had hypocomplementemia and cryoglobulinemia. Renal biopsy revealed membranoproliferative glomerulonephritis (MPGN), and she was diagnosed as having HCV-associated cryoglobulinemic MPGN. She declined interferon therapy at the time and was treated with antihypertensive medications as well as oral corticosteroid that were effective in reducing proteinuria. However, when the corticosteroid dose was reduced, proteinuria worsened. She began antiviral treatment with daclatasvir/asunaprevir (DCV/ASV). Clearance of HCV-RNA was obtained by 2 weeks and sustained, and liver function was normalized. In addition, microhematuria turned negative, proteinuria decreased, hypocomplementemia and estimated glomerular filtration rate were improved, whereas cryoglobulinemia persisted. She completed 24 weeks of therapy without significant adverse effects.

**Conclusion:**

In a case of HCV-associated cryoglobulinemic MPGN with renal dysfunction, DCV/ASV -based DAAs ameliorated microhematuria, proteinuria and renal function without significant side effects.

## Background

Recently, several direct-acting antivirals (DAAs) have been approved for treating hepatitis C virus (HCV) infections. The emergence of DAAs dramatically changed HCV treatments with superior rates of sustained viral response (SVR) and fewer side effects than the conventional interferon-based therapy. Information about the effects of DAAs on extra-hepatic manifestations, such as HCV-associated glomerulonephritis, skin lesions and neuropathy, is limited. Sise et al. reported that sofosbuvir-based DAAs therapy reduced proteinuria and increased estimated glomerular filtration rate (eGFR) in 3 of 7 patients with HCV-associated glomerulonephritis [1], although, sofosbuvir is contraindicated in patients with an eGFR of less than 30 ml/min/1.73 m^2^ Saadoun et al. reported that sofosbuvir plus ribavirin were effective in 4 of 5 patients with HCV-associated glomerulonephritis [2], although ribavirin is generally contraindicated in patients with a creatinine clearance of less than 50 mL/min. Thus information on the efficacy and safety in patients with impaired renal function is limited. Daclatasvir (DCV) is a NS5A replication complex inhibitor, and asunaprevir (ASV) is a selective NS3 protease inhibitor [3]. Both DCV and ASV have demonstrated robust antiviral activity against HCV [3, 4], and the combination of DCV and ASV as all-oral therapy was approved in Japan for the treatment of HCV hepatitis genotype 1b. This is an important option, especially for patients with renal impairments, since both DCV and ASV have minimal renal excretion and no dosage adjustment of DCV and ASV is generally required for patients with any degree of impaired renal function.

We demonstrate that novel DAA therapy with DCV/ASV improved renal function, microhematuria and proteinuria in a case of HCV-associated cryoglobulinemic membranoproliferative glomerulonephritis (MPGN).

## Case presentation

A 55-year-old Japanese woman, with no prior history of blood transfusion, drug addiction, or having tattoos was diagnosed as having an HCV infection at the local clinic where she had been treated for hypertension. At the age of 61, she complained of exanthems in both her lower extremities and arthralgia in her knees. She had purpura, and skin biopsy revealed leukocytoclastic angitis. Then, she was referred to a nephrologist since she had microscopic hematuria, proteinuria and renal dysfunction. At the time of referral, her blood pressure was 127/80 mmHg, her hight was 144.5 cm and her weight was 63.0 kg. She exhibited edema in her face and both lower extremities. Fresh purpura was present on her legs. Laboratory values at the time of referral were as follows: white blood cells 4470/μL, hemoglobin 11.4 g/dL, platelets 194 × 10^3^ μL, hepaplastin test 120%, total protein 7.2 g/dL, albumin 4.2 g/dL, cholinesterase 280 U/l, lactate dehydrogenase 155 U/L, glutamate oxaloacetate transaminase 33 U/ml, glutamate pyruvate transaminase 32 U/L, γ-glutamyl trans peptidase 40U/L, blood urea nitrogen 21 mg/dL, creatinine 1.2 mg/dL, eGFR 36.1 ml/min/1.73 m^2^, C-reactive protein 0.2 mg/dL, MPO-ANCA <10 EU, PR3-ANCA <10 EU, anti nuclear antibody × 40 (normal limit < ×40), rheumatoid factor 436 (0–15) IU/ml, IgG 1752 (1100–1700) mg/dl, IgA 169 (110–410) mg/dl, IgM 228 (46–260) mg/dl, C3 51 (65–135) mg/dl, C4 3 (13–35) mg/dl, CH50 < 10 (23–46) U/ml and cryoglobulin was positive. Urinalysis showed hematuria (+++): sediment red blood cells >100/high power field (HPF) and proteinuria 0.78 g/day.

**Fig. 1:**
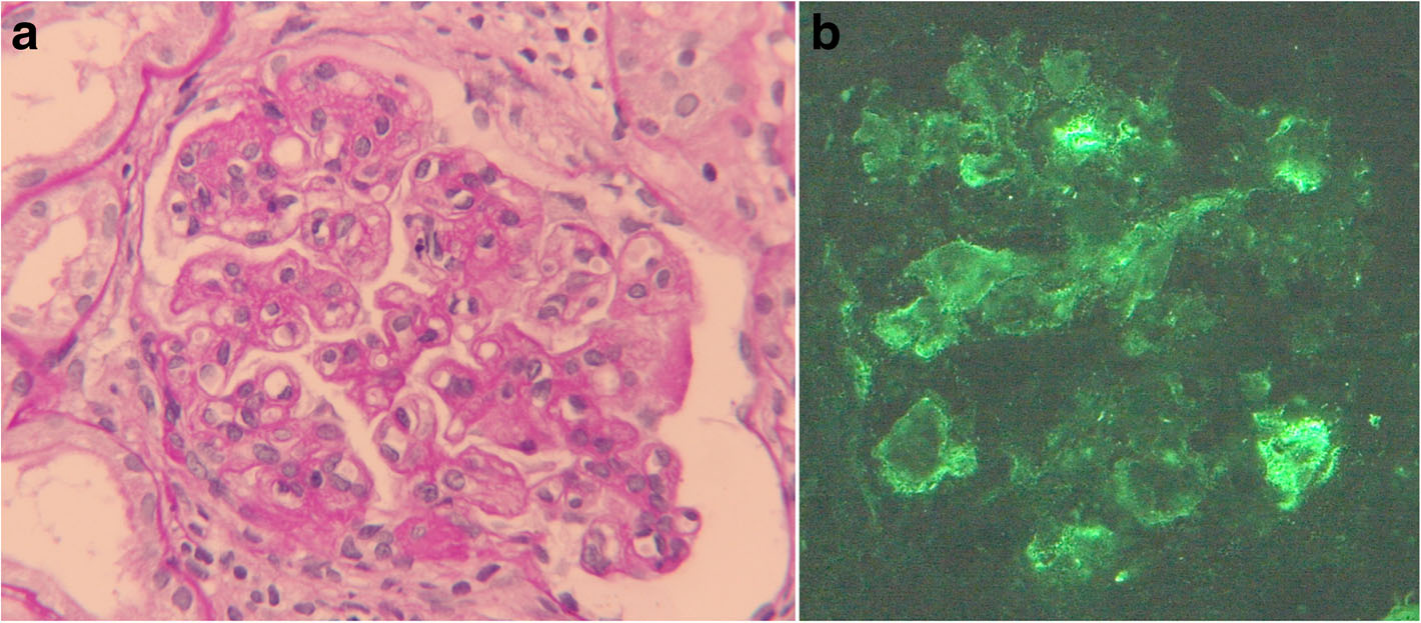
**a** Light microscopy findings of kidney biopsy. Periodic acid-Schiff stain reveals mesangial hypercellularity, lobular accentuation and double contour of the basement membrane. (original magnification, ×400). **b**. Immunofluorescent staining of IgM was positive along the capillary loop. (original magnification, ×400)

A renal biopsy was performed. On light microscopy, 30 glomeruli were observed and 4 glomeruli exhibited global sclerosis. Many other glomeruli exhibited mesangial hypercellularity, lobular accentuation and double contour of basement membrane (Fig. 1a). Pseudothrimbi and tubuloreticular inclusions were not observed. Immunofluorescent analysis showed a positive fringe pattern for IgG, IgM (Fig. 1b), C3. Staining for IgA was negative. Electron microscopy was not performed. Therefore, her case was diagnosed as having MPGN. Since, she was HCV positive, and had hypocomplementemia and cryoglobulinemia, she was diagnosed as having HCV-related cryoglobulinemic MPGN.

She was then referred to a hepatologist. Liver biopsy revealed chronic hepatitis and there were no signs of cirrhosis. She was diagnosed with chronic hepatitis due to HCV. At the time, she declined interferon therapy because she was regularly seeing a psychiatrist and taking anti-anxiety medication and was concerned about depression as a side effect. She was treated with antihypertensive medications, including renin-angiotensin system blockers as well as oral corticosteroid which were effective in reducing proteinuria. However, proteinuria was exacerbated following the reduction of corticosteroid and often reached nephrotic levels. She began anti-viral therapy using DAAs. Because of renal dysfunction, DCV and ASV were selected. Variation in Y93/L31, which is related to DCV resistance, was negative. DCV (60 mg/day) and ASV (200 mg/day) were initiated. Clearance of HCV RNA was obtained by 2 weeks and sustained, and liver function was normalized. In addition, microhematuria turned negative, proteinuria decreased, hypocomplementemia and eGFR improved, whereas cryoglobulinemia persisted. Arthralgia and purpura were not active during the treatment. Data are summarized in Table 1. She completed 24 weeks of therapy without significant adverse effects.

**Table 1:** Laboratory data during DCV/ASV therapy

Data	Normal range							
Weeks		0	4	10	14	18	22	24
Creatinine	(0.4–0.7)	1.83	1.51	1.33	1.07	1.18	1.1	1.06
eGFR (mL/min/1.73 m^2^)		25.9	27.2	31.1	39.5	35.5	35.5	38.3
C3 (mg/dl)	(35–65)	56	86	87	96	95	99	94
C4 (mg/dl)	(13–35)	1	4	3	4	3	3	3
CH50 (U/mL)	(23–46)	10	17.2	17.4	39.6	31.9	32.7	29.1
Proteinuria (g/g・cre)	(<0.15)	1.94	0.62	0.16	1.38	0.65	0.93	0.13
Hematuria(/HPF)	(<5)	50	10.8	5.3	15.3	10.8	12.2	3.9
Cryoglobulin	(−)	+		+		+		+
GOT (U/L)	(13–33)	32	27	25	24	24	27	24
GPT (U/L)	(6–27)	32	23	23	22	21	19	20
γ-GTP (U/L)	(10–47)	63	51	32	26	24	24	22

*Abbreviations: eGFR* estimated glomerular filtration rate, *HPF* high power field, *GOT* glutamate oxaloacetate transaminase, *GPT* glutamate pyruvate transaminase, *γ-GTP* γ-glutamyl trans peptidase

## Discussion

To our knowledge, this is the first case report which demonstrates renoprotective effects of novel DAAs therapy with DCV/ASV in the case of biopsy proven HCV-related cryoglobulinemic MPGN. This was a case of HCV chronic hepatitis with concomitant MPGN, arthralgia and purpura due to cryoglobulinemia. Multi-drug antihypertensive therapy including renin-angiotensin system blockade and oral corticosteroid therapy had been used but proteinuria was persistent and she had chronic kidney disease stage 3B renal impairment. Then DCV/ASV-based DAA therapy was employed, and a prompt antiviral response, normalization of liver function and subsequent improvement of eGFR, hypocomplementemia, hematuria and proteinuria were obtained without significant side effects. In contrast, cryoglobulinemia was persistent. However, this is consistent with a previous report showing that cryoglobulinemia persisted even after the clearance of HCV virus [5].

HCV infection leads to chronic liver disease, as well as extra-hepatic manifestations which usually derive from HCV-associated cryoglobulinemic vasculitis. The relationship between HCV and MPGN was initially described in 1993 [6]. At present, it is known that MPGN accompanied with cryoglobulinemia is the most common form of HCV-related glomerulonephropathy [7], and it is often accompanied with hypocomplementemia, rheumatoid factor and cryoglobulinemia [5]. Interferon therapy was effective in reducing proteinuria [8]; however, its effect was limited and dependent on the achievement of SVR [9], and recurrence of proteinuria was very common with the recurrence of viremia [10]. Therefore, proteinuria was often persistent and renal insufficiency was progressive. Combined antiviral therapy using pegylated interferon and ribavirin significantly improved treatment outcome. However, ribavirin is contraindicated if eGFR is less than 50 mL/min/1.73 m^2^, and the patients who were not able to obtain SVR usually did not receive the benefit. Treatment guidelines suggest that patients with HCV and mixed cryoglobulinemia with nephrotic proteinuria, or evidence of progressive kidney disease, or an acute flare of cryoglobulinemia, will benefit from treatment with plasmapheresis, rituximab, or cyclophosphamide, in conjunction with intravenous methylprednisolone, and concomitant antiviral therapy [11, 12]. Rituximab contributed to improved renal outcome, however, the effects were often transient and repeated treatment was required [13, 14].

Recent induction of DAA therapy has dramatically changed HCV treatment, with superior rates of SVR and fewer side effects. Therefore, improvements in HCV-associated glomerulonephritis are also expected. However, we are awaiting further evidences showing efficacy and safety in patients with impaired renal function. Tsuge et al. reported a case of improved eGFR with DCV/ASV therapy in a case of HCV cirrhosis, however, renal histology in this case is not available [15].

## Conclusion

DCV/ASV-based DAA therapy ameliorated microhematuria, proteinuria and renal function in a case of HCV-related cryoglobulinemic MPGN with renal dysfunction. Since this is a case report, further evidence for the efficacy and safety in patients with renal impairments is needed.

## Abbreviations

ASV: Asunaprevir
DAA: Direct-acting antivirals
DCV: Daclatasvir
eGFR: Estimated glomerular filtration rate
HCV: Hepatitis C virus
HPF: High power field
MPGN: Membranoproliferative glomerulonephritis
SVR: Sustained viral response
